# IL-36α Exerts Pro-Inflammatory Effects in the Lungs of Mice

**DOI:** 10.1371/journal.pone.0045784

**Published:** 2012-09-20

**Authors:** Ravisankar A. Ramadas, Susan L. Ewart, Yoichiro Iwakura, Benjamin D. Medoff, Ann Marie LeVine

**Affiliations:** 1 Center for Immunology and Inflammatory Diseases, Harvard Medical School, Massachusetts General Hospital, Boston, Massachusetts, United States of America; 2 Pulmonary and Critical Care Unit, Massachusetts General Hospital, Boston, Massachusetts, United States of America; 3 Department of Large Animal Clinical Sciences, Michigan State University, East Lansing, Michigan, United States of America; 4 Center for Experimental Medicine and Systems Biology, University of Tokyo, Tokyo, Japan; 5 Department of Pediatrics and Communicable Diseases, University of Michigan, Ann Arbor, Michigan, United States of America; French National Centre for Scientific Research, France

## Abstract

Interleukin (IL-) 36 cytokines (previously designated as novel IL-1 family member cytokines; IL-1F5– IL-1F10) constitute a novel cluster of cytokines structurally and functionally similar to members of the IL-1 cytokine cluster. The effects of IL-36 cytokines in inflammatory lung disorders remains poorly understood. The current study sought to investigate the effects of IL-36α (IL-1F6) and test the hypothesis that IL-36α acts as a pro-inflammatory cytokine in the lung *in vivo*. Intratracheal instillation of recombinant mouse IL-36α induced neutrophil influx in the lungs of wild-type C57BL/6 mice and IL-1αβ^−/−^ mice *in vivo*. IL-36α induced neutrophil influx was also associated with increased mRNA expression of neutrophil-specific chemokines CXCL1 and CXCL2 in the lungs of C57BL/6 and IL-1αβ^−/−^ mice *in vivo*. In addition, intratracheal instillation of IL-36α enhanced mRNA expression of its receptor IL-36R in the lungs of C57BL/6 as well as IL-1αβ^−/−^ mice *in vivo*. Furthermore, in vitro incubation of CD11c^+^ cells with IL-36α resulted in the generation of neutrophil-specific chemokines CXCL1, CXCL2 as well as TNFα. IL-36α increased the expression of the co-stimulatory molecule CD40 and enhanced the ability of CD11c^+^ cells to induce CD4^+^ T cell proliferation *in vitro*. Furthermore, stimulation with IL-36α activated NF-κB in a mouse macrophage cell line. These results demonstrate that IL-36α acts as a pro-inflammatory cytokine in the lung without the contribution of IL-1α and IL-1β. The current study describes the pro-inflammatory effects of IL-36α in the lung, demonstrates the functional redundancy of IL-36α with other agonist cytokines in the IL-1 and IL-36 cytokine cluster, and suggests that therapeutic targeting of IL-36 cytokines could be beneficial in inflammatory lung diseases.

## Introduction

The Interleukin 1 (IL-1) cytokine cluster consists of a network of agonist, antagonist and receptor molecules that are expressed in a variety of immune and structural cells [1]. Since its initial identification as the human leukocytic pyrogen that causes fever [2], IL-1 has emerged as highly organized facilitator of early immune events in a plethora of inflammatory diseases [3]. The role of IL-1 cytokines in the development of acute and chronic inflammatory disorders of the lung has been extensively documented and mechanistically characterized in human patients and in animal models of human lung disorders. For example, increased levels of IL-1 protein or mRNA, reflective of increased IL-1 cytokine activity, has consistently been observed in tissues from human patients with inflammatory lung disorders such as asthma [4]–[7], acute lung injury [8]–[10] and pulmonary fibrosis [11]. Similarly, studies from animal models have demonstrated that IL-1 cytokines such as IL-1α and IL-1β play critical roles in the pathogenesis of asthma [12]–[14], chronic obstructive pulmonary disease [15], [16], acute lung injury [17] and pulmonary fibrosis [18]–[20]. In pulmonary infection models, IL-1 promoted or dampened bacterial clearance from the lungs depending on the bacterial pathogen [21]–[23]. While IL-1 induced the recruitment of neutrophils and pulmonary inflammation during the early stages of influenza infection in mice [24], it was critical in conferring protective immunity and enhancing survival of mice during the later stages of the disease [24]–[26].

In the last decade, genes encoding a novel cytokine cluster with structural and functional similarities to interleukin 1 (IL-1) were discovered and mapped to chromosome 2 in humans and mice [27]–[31]. These cytokines, initially termed IL-1 family members 5–10 (IL1F5– IL1F10) have been recently reclassified according to an updated cytokine nomenclature scheme [32]. This novel IL-1 like cytokine cluster comprise a collection of agonists IL-36α (IL-1F6), IL-36β (IL-1F8), IL-36γ (IL-1F9), an antagonist IL-36Ra (IL-36Ra), a nuclear factor IL-37 (IL-1F7) and a functional heterodimeric receptor complex consisting of a novel receptor IL-36R (IL-36R) and the adapter molecule IL-1 receptor accessory protein (IL-1RAcP) [33]. In addition, IL-38 (IL-1F10) has recently been reported to act as a partial antagonist for IL-36R [34]. The organization and function of this novel IL-1 like cytokine cluster is strikingly similar to that of the classical IL-1 cytokine cluster, which consists of agonists (IL-1α, IL-1β) and an antagonist (IL-1Ra) which binds to a functional heterodimeric receptor complex containing IL-1 receptor I (IL-1R1) and IL-1RAcP [35]. Both the classical and novel IL-1 cytokine cluster agonists induce the activation of nuclear factor kappa B (NF-κB) and mitogen associated protein kinase (MAPK) to initiate pro-inflammatory pathways [31], [35]. While the role of classical IL-1 cytokines have been extensively characterized in the pathogenesis of inflammatory disorders [35], [36], the disease-relevant functions of the novel IL-1 like cytokines (IL-36 cytokines) remain poorly characterized.

We have previously demonstrated that IL-36γ is constitutively expressed and inducibly upregulated in the lungs of mice following allergic inflammation [37], [38]. Airway epithelial cells express IL-36α and IL-36γ [38], [39], and the mRNA expression of IL-36α and IL-36γ in airway epithelial cells is increased in response to several inflammatory stimuli [39], [40]. IL-36α and IL-36γ directly act on IL-36R expressing lung fibroblasts to activate NF-κB and MAPK pathways [39]. We have demonstrated that intratracheal instillation of recombinant IL-36γ increased airway hyperresponsiveness, induced neutrophil influx and neutrophil-specific chemokine production the lungs *in vivo* [38]. We also demonstrated that IL-36γ activated NF-κB in whole lung tissue and alveolar macrophage cell lines [38]. Reports from other groups have demonstrated that the mRNA expression of IL-36α and IL-36γ were also increased in human bronchial epithelial cells exposed to *Pseudomonas aeruginosa* [40] and in recurrent respiratory papillomas [41]. Recent reports have also demonstrated the critical role of IL-36α, IL-36Ra and IL-36R in the development of psoriasis [42]–[45], and that a single amino acid mutation in IL-36Ra can lead to unchecked cytokine production and pustular psoriasis [46]. Accumulating evidence thus points to the existence of a novel, underexplored signaling pathway in inflammatory diseases, driven by the IL-36 cytokine cluster.

Despite the recent progress in elucidating the role of IL-36 cytokines in inflammatory diseases, the *in vivo* role of IL-36α has not been extensively investigated in inflammatory lung disorders. Since multiple cytokines contribute to shaping the overall immune response in inflammatory disorders, it is necessary to characterize the proportional contribution and functional redundancy of individual cytokines to comprehensively understand disease pathogenesis and devise therapeutic strategies. Since it has been demonstrated that the mRNA expression of IL-36α in the airway epithelial cells are increased in response to multiple pro-inflammatory stimuli [39], we hypothesized that the presence of IL-36α in the lungs may induce pulmonary inflammation *in vivo*. Here we demonstrate that intratracheal instillation of recombinant mouse IL-36α induces neutrophil influx and the production of neutrophil-specific chemokines in wild-type mice as well as mice that lack IL-1α and IL-1β. Furthermore, we also demonstrate that IL-36α acts directly on CD11c^+^ cells to induce the expression of neutrophil-specific chemokines, T cell costimulatory molecules and the enhancement of CD4^+^ T cell proliferation. Finally, we also demonstrate that IL-36α activates NF-κB in mouse macrophages. These results suggest that IL-36α acts as a pro-inflammatory cytokine in the lung, and that IL-36α could increase inflammatory responses in disease conditions which involve release of IL-36α into the lungs.

## Materials and Methods

### Mice and Procedures

Six to eight week old mice (C57BL/6, C3H/HeJ and OTII TCR transgenic strains) were purchased from Jackson Laboratory (Bar Harbor, ME). Mice deficient in IL-1α and IL-1β (IL-1α/β^−/−^) were obtained from Dr. Yoichiro Iwakura [47] and backcrossed to C57BL/6 background. All mice were housed or bred in specific pathogen free facility at Massachusetts General Hospital. For intratracheal instillations, mice were anesthetized with a mixture of xylazine (8 mg/kg) and ketamine (45 mg/kg). Recombinant protein preparation or phosphate buffered saline (PBS) controls in a 50 µl volume were intratracheally (i.t.) instilled using a non-invasive method. Mice were euthanized with an overdose of pentobarbital, and tissues were collected at indicated time points following the procedure. For cell isolation protocols that do not require isolation of lung tissue, mice were euthanized by carbon-dioxide asphyxiation.

### Ethic Statement

Mice were housed in a temperature controlled specific-pathogen free facility at Massachusetts General Hospital, and provided unrestricted access to food and water. All protocols involving the use of mice were approved by Subcommittee on Research Animal Care at Massachusetts General Hospital.

### Generation of Recombinant IL-36α

A cDNA clone containing full length mouse *Il36α (Il1f6*, Clone ID: MMM1013-99829107) was obtained (Open Biosystems). A PCR product encoding a fusion protein comprised of an N-terminal FLAG tag followed by the full length coding sequence of *Il36α* was generated from this clone using the following restriction-site engineered primers: Forward 5′-CGGAATTCCgattacaaggatgacgatgacaagAATAAGGAGAAAGAACTAAGAG-3′ and reverse 5′-AAGGAAAAAAGCGGCCGCTTAATGTACCACAATCATCTC-3′. The PCR product and pETDuet-1 vector (Clontech) were digested with EcoRI and NotI. The digested PCR product was cloned in-frame with the Histidine purification tags into the pETDuet-1 vector. The ligation reactions were transformed into *E. coli* and positive clones were identified. Expression of His_FLAG_IL-F6 fusion protein was induced in positive clones by addition of 1 mM Isopropyl β-D-1-thiogalactopyranoside (IPTG) to log-phase bacterial cultures for 4 h. Following induction, bacterial pellets were frozen at −20°C until protein isolation. IL-36α was purified from bacterial lysate by Ni^2+^ column chromatography (B-PER 6xHis Fusion Protein Kit, Pierce), and dialyzed against PBS. Purified, dialyzed IL-36α was treated with enterokinase (EK-Max, Invitrogen) to cleave His_FLAG tags from IL-36α protein. Enterokinase was removed from the tag-cleaved IL-36α preparation by treating with EK-Away (Invitrogen), according to the manufacturer’s protocols. The purified, tag-cleaved, enterokinase-removed recombinant IL-36α preparation was dialyzed and the dialyzed preparation was passed through a Ni^2+^ chromatography column. Cleaved tags bound to the column and the tag-free IL-36α preparations recovered from the flow-through were dialyzed against PBS, treated with polymyxin-B agarose beads (Detoxi-Gel, Pierce) to remove LPS contamination. LPS concentration in the purified IL-36α preparation was <0.01 EU/µg protein, as measured by Limulus Amebocyte Lysate assay (Lonza). Tag-removed, polymyxin-treated recombinant mouse IL-36α was used for *in vitro* and *in vivo* studies.

### Coomassie Staining

Ten µg of IL-36α was separated on a SDS-polyacrylamide gel under reducing and denaturing conditions. The gel was soaked in Fairbank’s Coomassie solution A (25% isopropanol, 10% acetic acid and 0.05% Coomassie R), microwaved until before boiling and shaken for 10 minutes at room temperature to stain the gel. The stain was drained, and the gel was soaked in distilled water and microwaved multiple times and washed in water to destain the gel.

### Western Immunoblotting

Increasing amounts of IL-36α (5, 10 and 20 ng) were separated on a SDS-polyacrylamide gel under reducing and denaturing conditions. Proteins were transferred to polyvinylidene fluoride membranes, probed with rat anti-mouse IL-36α antibody (R&D Systems, Catalog No. MAB2297) and detected with a horseradish peroxidase conjugated goat anti-rat secondary antibody (Abcam) and ECL Plus chemiluminescent detection system (Amersham).

### Tissue Collection and Analysis

Mice were euthanized with an overdose of pentobarbital, and the treacheas were catheterized. Lungs were lavaged three times with 1 mL of ice cold-PBS. Bronchoalveolar lavage (BAL) fluid was centrifuged at 1,500 rpm for 15 minutes and the supernatants were stored at −80°C. Cells in the pellet were used to calculate total cell numbers (Cellometer, Nexelcom), as well as differential cell counts on cytospun slides stained with Hema-3 White Cell Differential Staining Kit (Fisher). More than 100 cells were counted per sample by light microscopy to calculate differential cell counts. Upper right lobe of the lung was collected for RNA analysis, and the left lung was inflation fixed with 10% neutral buffered formalin for histological analysis.

### RNA Isolation and Quantitative Real-time PCR

Total RNA was isolated from lung tissue using Trizol (Invitrogen) and cell cultures using RNeasy (Qiagen) and treated with Amplification grade DNaseI (Invitrogen) according to manufacturer’s protocols. Equal concentrations of DNaseI treated RNA was reverse transcribed into cDNA using Taqman Reverse Transcription reagents (Applied Biosystems) and used as input for quantitative real-time PCR using Power SYBR Green kit (Applied Biosystems). Primers used in real-time PCR analyses were designed using the mouse qPrimerdepot (http://mouseprimerdepot.nci.nih.gov/). Cycle threshold (C_T_) values obtained from the assays were analyzed and reported as copies of target genes per copy of GAPDH, a housekeeping gene.

### Measurement of Protein Levels in the BAL Fluid

Cytometric bead array Flex-kits (BD Biosciences) for IL-1α, IL-1β, TNFα and CXCL1 were used according to manufacturer’s protocols to evaluate the protein levels in the BAL fluid recovered from the mice.

### Measurement of Lung Function in Mice

Six to eight week old mice were intratracheally administered 10 µg IL-36α or 50 µl PBS, and lung function measurements were performed 24 h later using the Flexivent system (Scireq, Montreal, Quebec, Canada), as described before [38]. Briefly, mice were anesthetized with an intraperitoneal injection of xylazine (12 mg/kg) and pentobarbital (70 mg/kg). The trachea of anesthetized mice was cannulated and the mice were ventilated with 6 ml/kg tidal volume at 150 breaths per minute. To suppress spontaneous breathing during measurement of lung function, mice were intraperitoneally injected with pancuronium bromide (2 mg/kg). Incremental doses of neublized methacholine were used to determine total lung resistance and compliance according to the Snapshot-150 perturbation provided by the Flexivent equipment. Thirteen data points were collected for each methacholine dose, and only data with a coefficient of determination greater than 0.95 were included in analyses. The survival of mice during the procedure was simultaneously monitored using electrocardiograms.

### Isolation and Culture of CD11c^+^ Cells

Splenic CD11c^+^ cells were isolated from wildtype C57BL/6 using magnetic separation according to manufacturer’s (Miltenyi Biotec) protocol. Briefly, single cell suspensions prepared from spleens of wildtype C57BL/6 mice were incubated with red blood cell lysis solution (Invitrogen) to lyse RBCs. The resulting cell suspensions were incubated with mouse anti-CD11c microbeads (Miltenyi Biotec), and CD11c^+^ cells were isolated by two rounds of positive selection to enhance purity. The purity of isolated CD11c^+^ cells were routinely >90%. The isolated CD11c^+^ cells were resuspended in RPMI-1640 media containing 10% heat inactivated fetal bovine serum (FBS), L-glutamine and 1% penicillin-streptomycin. According to the experiment, aliquots of resuspended cells were mixed with various concentrations of IL-36α (0.1 µg/mL, 1.0 µg/mL or 10.0 µg/mL) or an equivalent volume of PBS. For RNA isolation, cells mixed with IL-36α or PBS were plated at a concentration of 2.5×10^5^ cells per well in a 48-well plate in a volume of 250 µl. The cells were incubated for 2 hours to adhere. The media was gently removed from the plates by pipetting, and the cells were washed once with sterile, cold PBS. RNA was isolated from the adherent cells using RNeasy RNA isolation kit (Qiagen) according to manufacturer’s protocols. In some experiments, the media containing IL-36α was removed from the wells 2 hours after incubation, replaced with RPMI-1640 growth media and allowed to incubate for an additional 22 hours before evaluating cell surface expression of co-stimulatory molecules by flow cytometry.

### Isolation and Culture of CD4^+^ T Cells

Spleens and pooled lymph nodes from wildtype C57BL/6 mice were used to isolate CD4^+^ cells using magnetic separation according to manufacturer’s (Miltenyi Biotec) protocol to a routine purity of >95%. Isolated CD4^+^ cells were stained with CFSE (Celltrace CFSE staining kit, Invitrogen) and resuspended in culture media. For CD4^+^ and CD11c^+^ co-culture studies, CD11c^+^ cells were isolated using magnetic separation protocols, mixed with the indicated concentrations of IL-36α, plated at a concentration of 1×10^5^ cells per well in a 96-well plate and allowed to adhere for 2 hours. Following incubation, media from the wells was removed by pipetting, cells were washed once with cold PBS, and 4×10^5^ CFSE-stained CD4^+^ T cells were incubated with the adherent CD11c^+^ cells for 96 hours. Following incubation for 96 h, cells were removed and analyzed by flow cytometry. To evaluate antigen-specific responses, isolated splenic CD11c^+^ cells were plated at a concentration of 1×10^5^ cells per well with indicated concentrations of IL-36α and 100 ng/mL OVA_323-339_ for 2 hours. Following incubation, media from the wells was removed by pipetting, cells were washed once with cold PBS, and 4×10^5^ CFSE-stained CD4^+^ T cells from OTII TCR transgenic mice were incubated with the adherent CD11c^+^ cells for 96 hours. Following incubation for 96 h, cells were removed and analyzed by flow cytometry.

**Figure 1:**
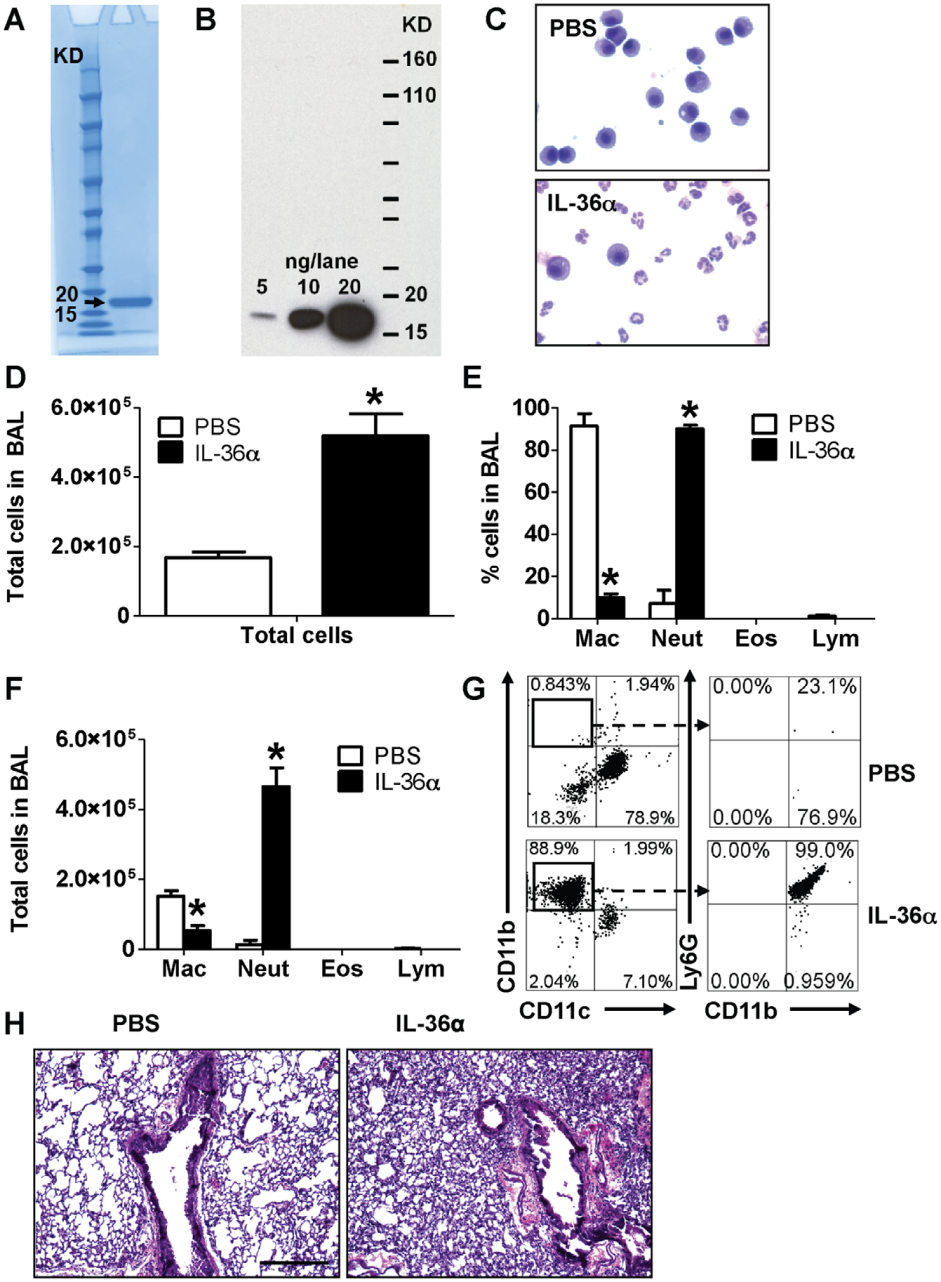
Intratracheal instillation of IL-36α induced neutrophil influx in the lungs of wild-type C57BL/6 mice. A) Coomassie blue stained gel demonstrating the purity of IL-36α preparation. Ten µg IL-36α was loaded on the lane. B) Western immunoblotting of IL-36α protein preparation detects a band around 18 KDa, the predicted molecular weight of mouse IL-36α. C) Cytospin preparations demonstrating neutrophil influx in the BAL fluid recovered from mice 24 h following a single i.t. instillation of PBS or 10 µg IL-36α. D) Total cell counts from BAL fluid recovered from mice 24 h following a single i.t. instillation of PBS or 10 µg IL-36α. E) Differential cell count percentages and F) Differential cell count numbers in the BAL fluid recovered from mice 24 h following a single i.t. instillation of PBS or 10 µg IL-36α. *Indicates significant differences (*P<0.05*) compared to PBS treated mice. Data represent mean±SEM from 4–5 mice/group. G) Flow cytometry on cells recovered from BAL fluid from mice 24 h following a single i.t. instillation of PBS or 10 µg IL-36α. A majority of cells from the IL-36α instilled lungs were CD11c^−^CD11b^+^Ly6G^+^ neutrophils. Depicted flow cytometry plots are representative of 4–5 mice/group. H) Hematoxylin & Eosin stained lung sections isolated from mice 24 h following a single i.t instillation of PBS or 10 µg IL-36α. Depicted sections are representative of 4–5 mice/group.

### Flow Cytometry

Cells isolated from BAL were incubated with Fc block, followed by incubation with fluorochrome conjugated antibodies against mouse CD11c, CD11b (BD Biosciences) or Ly6-G (R&D Systems) according to standard protocols. For CFSE-based cell proliferation studies, cells were collected and incubated with Fc block, followed by incubation with allophycocyanin-conjugated anti-CD4 (BD Biosciences) antibody. T cell proliferation responses were analyzed by gating on live CD4^+^ cells based on forward- and side-scatter properties. Flow cytometry data were acquired by an Accuri C6 flow cytometer (Accuri) and analyzed using CFlow software (Accuri) or Flowjo (Treestar).

### Isolation of Alveolar Macrophages from Naïve Mice

Naïve C57BL/6 mice were euthanized with an overdose of pentobarbital. Lungs of mice were lavaged three times with 1 mL of ice cold-PBS. Pooled BAL fluids from three mice were centrifuged at 1,500 rpm for 15 minutes. An aliquot of cells were used for cytospin analysis. RNA was isolated from the remaining cells using RNeasy RNA isolation kit (Qiagen). Isolated RNA was treated with DNaseI, reverse transcribed and the resulting cDNA was used as the input for PCR.

**Figure 2:**
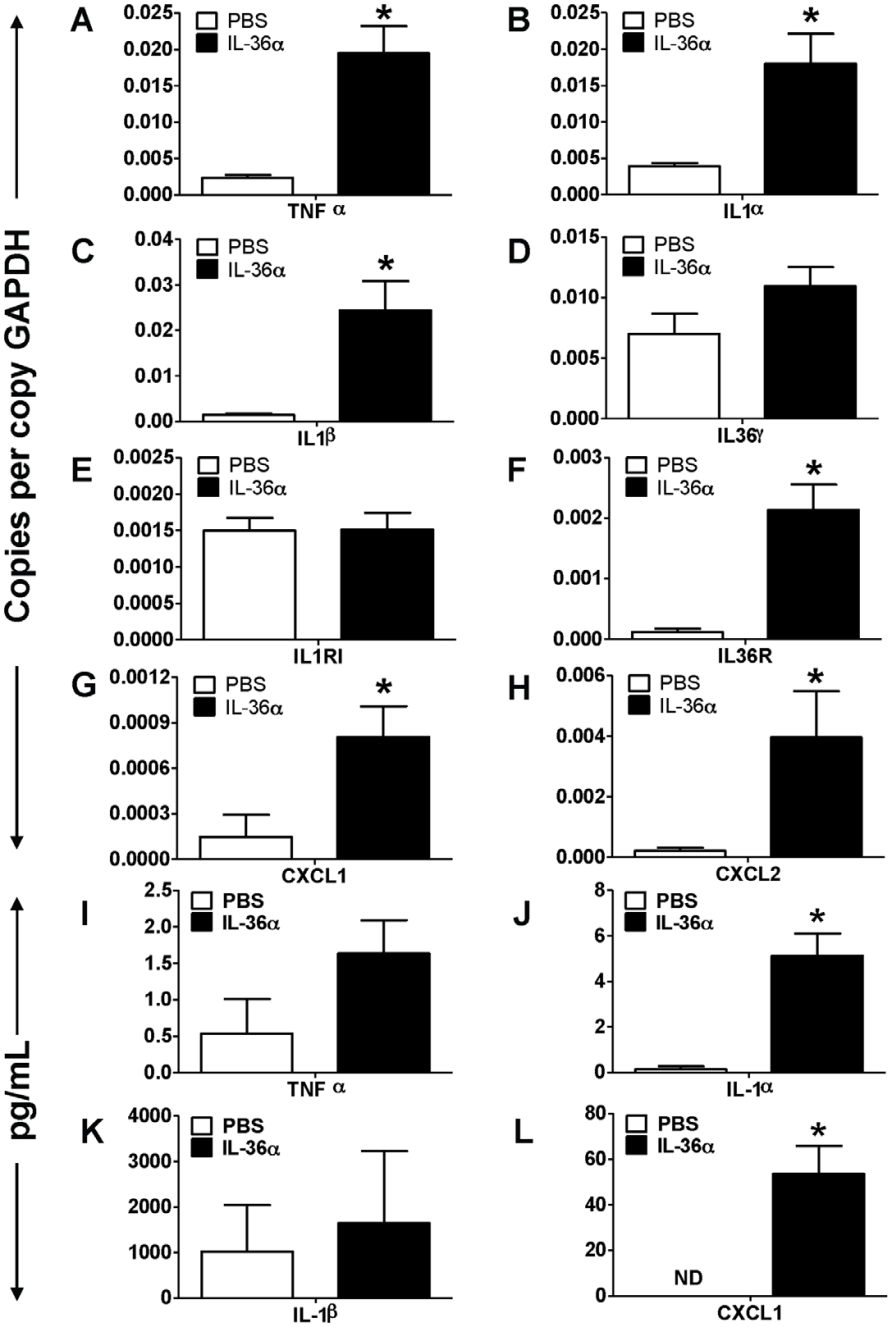
Intratracheal instillation of IL-36α increased the mRNA expression of proinflammatory mediators in the lungs of wild-type C57BL/6 mice. A–H) Transcript expression of early response cytokines (TNFα, IL-1α, IL-1β, IL-36γ), the classical IL-1 receptor IL-1R1, the novel IL-1 cytokine cluster receptor IL-36R and the neutrophil specific chemokines CXCL1 and CXCL2 in the lungs of mice 24 h following a single i.t instillation of PBS or 10 µg IL-36α. Transcript expression was evaluated by SYBR-Green based quantitative real-time PCR. I–K) Protein expression of TNFα, IL-1α, IL-1β and CXCL1 in the BAL fluid recovered from mice 24 h following a single i.t instillation of PBS or 10 µg IL-36α. Protein expression was quantified by multiplexed cytometric bead arrays. *Indicates significant differences (*P<0.05*) compared to PBS treated mice. Data represent mean±SEM from 4–5 mice/group.

### NF-κB Activation Assays in Mouse Macrophage Cell Lines

A mouse macrophage cell line (RAW-ELAM) stably transfected with the NF-κB responsive E-Selectin promoter driving the expression of enhanced green fluorescent protein [48] was used to investigate if IL-36α induced NF-κB activity in mouse macrophages. Cells were cultured in RPMI-1640 with 10% fetal bovine serum and 1% penicillin and streptomycin. One day before the experiments, cells were plated at a density of 5×10^5^ cells per well. Media was removed the day of the experiment, and IL-36α mixed with media was added to the cells and incubated for 6 h. After incubation, media was removed and cells were washed twice with ice-cold PBS. Cells were collected, and eGFP fluorescence reflective of NF-κB activity was measured using an Accuri C6 flow cytometer.

### Statistical Analysis

Data from BAL cell enumeration experiments and real-time PCR quantification on lung tissue were analyzed by two-tailed student’s t-test. Real-time PCR data from cell culture experiments were analyzed by two tailed student’s t-test. Lung resistance and compliance measurements were analyzed by two-way repeated measures ANOVA. Statistical significance was accepted at *P<0.05*.

**Figure 3:**
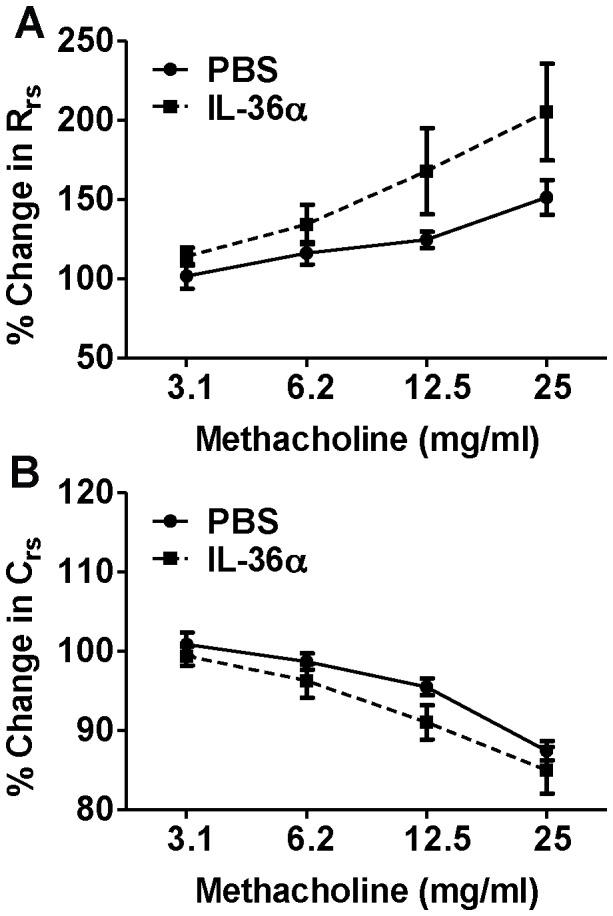
Intratracheal instillation of IL-36α does not induce airway hypresponsiveness. A) Total lung resistance and B) lung compliance were not significantly different in the lungs of IL-36α challenged mice compared to PBS controls. Airway responses in mice were measured using invasive plethysmography 24 h following i.t instillation of IL-36α or PBS. Data presented are percentage changes from baseline (0mg/mL) measurements. Data represent mean±SEM from 5–7 mice/group.

## Results

### IL-36α Induces Neutrophil Influx in the Lungs of Mice

We and others have previously demonstrated that IL-36γ is predominantly expressed in airway epithelial cells and that i.t. instillation of IL-36γ induces neutrophil influx in the lungs of mice [38], [39]. IL-36α and IL-36γ mRNA expression levels are increased in airway epithelial cells in response to various stimuli [39]. To investigate if IL-36α induces proinflammatory responses in the lung *in vivo*, we generated pure recombinant murine IL-36α protein (Fig. 1A & 1B). A single i.t. instillation of 10 µg IL-36α induced neutrophil influx (Fig. 1C) in the lungs of wild-type C57BL/6 mice 24 h following instillation. As expected, the majority of cells in the lungs of PBS instilled mice were macrophages (Fig. 1C). The total number of cells (Fig. 1D), percentage of neutrophils (Fig. 1E) and the total number of neutrophils (Fig. 1F) were significantly increased in the lungs of mice that received i.t. IL-36α compared to mice that received PBS. Flow cytometric evaluation of BAL cells also revealed that majority of cells recovered from BAL fluid of IL-36α instilled mice were CD11c^−^CD11b^+^Ly6G^+^ neutrophils, while a majority of cells recovered from the BAL fluid of PBS instilled mice were CD11c^+^CD11b^−^Ly6G^−^ cells, reflective of macrophages (Fig. 1G). Examination of Hematoxylin & Eosin stained lung sections revealed significant cellular infiltration in the lungs of IL-36α instilled mice (Fig. 1H) compared to the lungs of mice that received PBS. While we used polymyxin-treated, highly pure IL-36α, to ensure that the results we obtained are not due to trace amounts of contaminating LPS from the bacteria-derived IL-36α, we performed the same set of experiments in C3H/HeJ mice, which are unresponsive to LPS due to a spontaneous loss-of-function mutation in Toll like receptor 4 [49]. It has been previously demonstrated that LPS does not induce neutrophil influx or the production of neutrophil-specific chemokines CXCL1 and CXCL2 in the lungs of C3H/HeJ mice [50]. In the current study, a single i.t. instillation of 10 µg IL-36α induced neutrophil influx into the lungs (Fig. S1A), increased the total number of cells (Fig. S1B), percentage of neutrophils (Fig. S1C) and total number of neutrophils (Fig. S1D) in the lungs as assessed from BAL fluid recovered from C3H/HeJ mice 24 h following instillation. Macrophages were the main cell types present in the BAL fluid of PBS instilled C3H/HeJ mice. These results strongly suggest that the neutrophil influx observed in the lungs of mice is due to the agonist activity of IL-36α and not due to contaminants in the protein preparation. Taken together, these data demonstrate that neutrophil influx is the predominant response to overproduction of IL-36α in the lungs.

**Figure 4:**
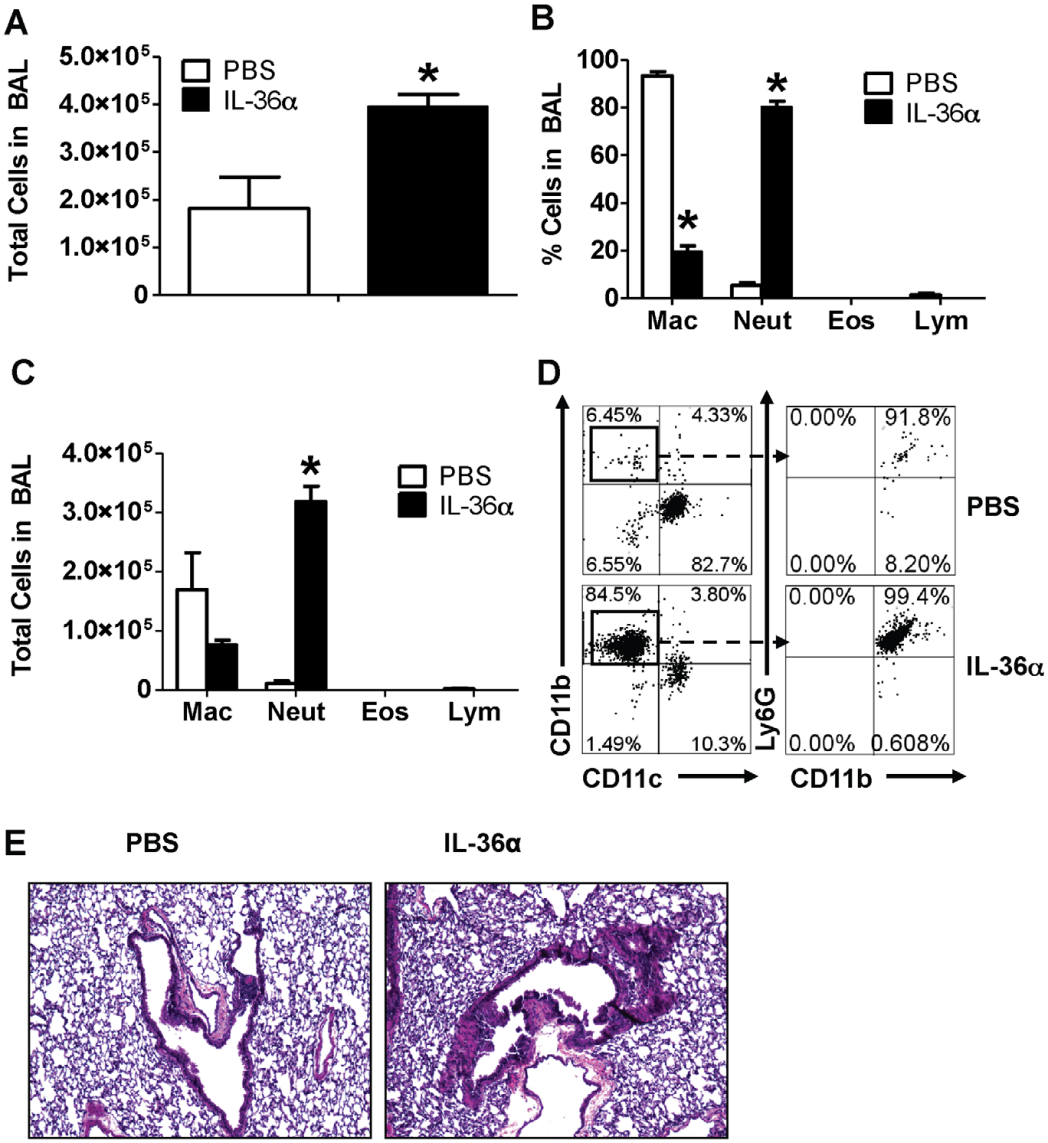
Intratracheal instillation of IL-36α induced neutrophil influx in the lungs of IL-1αβ^−/−^ mice. A) Total cell counts from BAL fluid recovered from mice 24 h following a single i.t. instillation of PBS or 10 µg IL-36α. B) Differential cell count percentages and C) Differential cell count numbers in the BAL fluid recovered from mice 24 h following a single i.t. instillation of PBS or 10 µg IL-36α. *Indicates significant differences (*P<0.05*) compared to PBS treated mice. Data represent mean±SEM from 3–4 mice/group. D) Flow cytometry on cells recovered from BAL fluid from mice 24 h following a single i.t. instillation of PBS or 10 µg IL-36α. A majority of cells from the IL-36α instilled lungs were CD11c^−^CD11b^+^Ly6G^+^ neutrophils. Depicted flow cytometry plots are representative of 3–4 mice/group. H) Hematoxylin & Eosin stained lung sections isolated from mice 24 h following a single i.t instillation of PBS or 10 µg IL-36α. Depicted sections are representative of 3–4 mice/group.

### IL-36α Induces the Expression of Early Response Cytokines and Neutrophil Specific Chemokines

To determine IL-36α driven molecular mechanisms that induce neutrophil influx into the lungs, we collected lung tissue from wildtype C5BL/6 mice 24 h following a single i.t. instillation of IL-36α and profiled the mRNA expression of selected cytokines, chemokines as well as agonists and receptors in the IL-1 cytokine network. The mRNA expression of the early response cytokine TNFα was significantly increased in the lungs of mice which received i.t. instillation of IL-36α, compared to saline controls (Fig. 2A). Similarly, the mRNA expression of IL-1α (Fig. 2B) and IL-β (Fig. 2C) were also significantly increased in the lungs of IL-36α instilled mice, while the expression of IL-36γ was unaltered (Fig. 2D). We did not detect the mRNA expression of other agonist IL-36 cytokines in the lungs under the conditions used (data not shown). While the mRNA expression of the classical IL-1 receptor (IL-1RI) was unchanged (Fig. 2E), the mRNA expression of the IL-36R, the receptor for IL-36α and IL-36γ, was significantly increased in the lungs of IL-36α instilled mice (Fig. 2F). In addition, the mRNA expression of the neutrophil recruiting chemokines CXCL1 (Fig. 2G) and CXCL2 (Fig. 2H) were also significantly increased in the lungs of IL-36α instilled mice compared to PBS controls. The protein levels of IL-1α (Fig. 2J) and CXCL1 (Fig. 2L) were significantly increased in the BAL fluid recovered from IL-36α instilled mice, however the protein levels of TNFα and IL-1β were not significantly increased. These results suggest that by inducing the expression of its receptor (IL-36R), IL-36α may potentially contribute to the initiation of a feedback signaling loop to enhance proinflammatory responses. Furthermore, these results also suggest that IL-36α acts on structural or immune cells in the lung to induce the production of CXCL1 and CXCL2, leading to neutrophil influx in the lungs.

**Figure 5:**
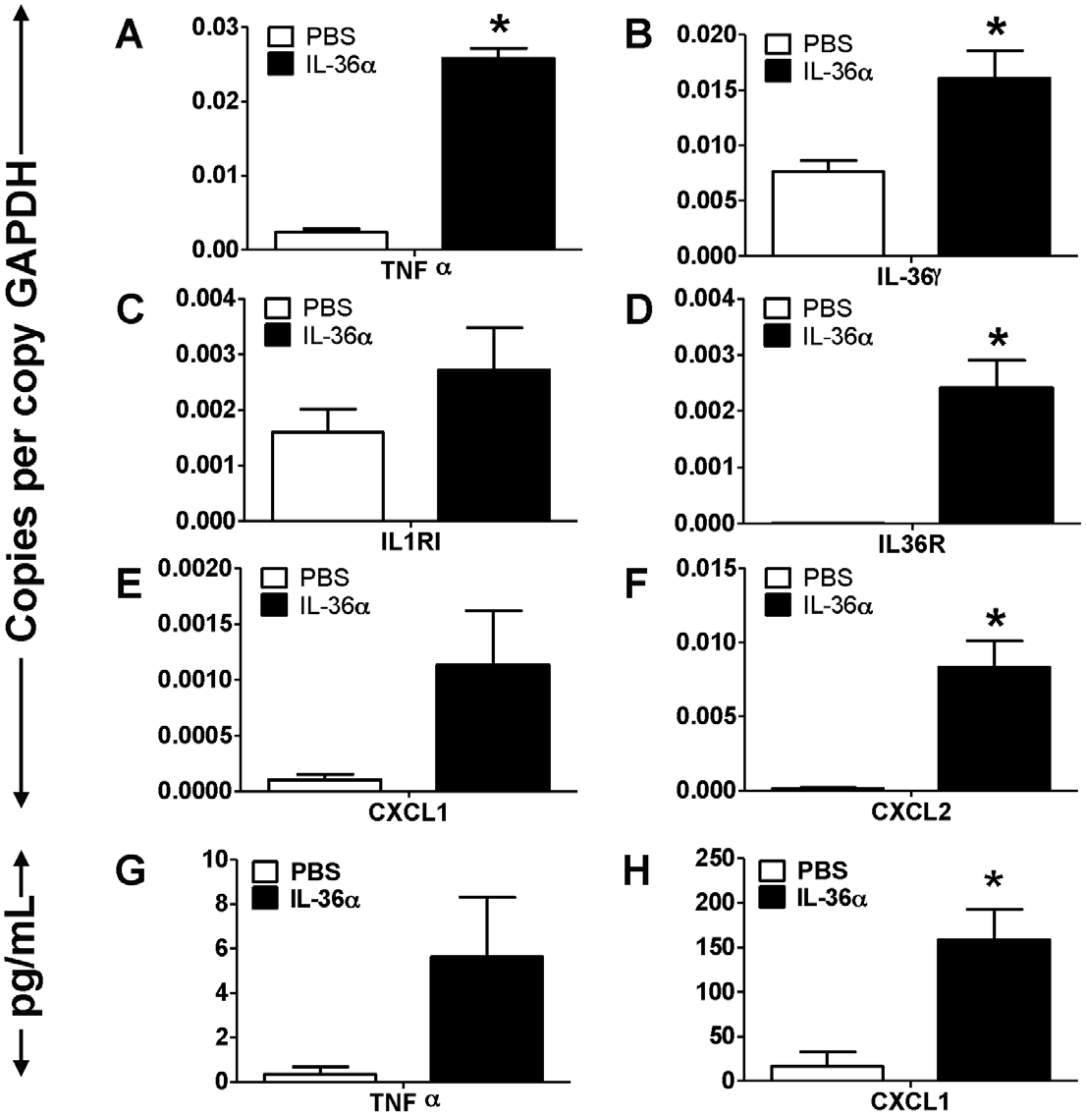
Intratracheal instillation of IL-36α increased the mRNA expression of proinflammatory mediators in the lungs of IL-1αβ^−/−^ mice. A–F) Transcript expression of early response cytokines (TNFα and IL-36γ), the classical IL-1 receptor IL-1R1, the novel IL-1 cytokine cluster receptor IL-36R and the neutrophil specific chemokines CXCL1 and CXCL2 in the lungs of IL-1αβ^−/−^ mice 24 h following a single i.t instillation of PBS or 10 µg IL-36α. Transcript expression was evaluated by SYBR-Green based quantitative real-time PCR. G–H) Protein expression of TNFα and CXCL1 in the BAL fluid recovered from IL-1αβ^−/−^ mice 24 h following a single i.t instillation of PBS or 10 µg IL-36α. Protein expression was quantified by multiplexed cytometric bead arrays. *Indicates significant differences (*P<0.05*) compared to PBS treated mice. Data represent mean±SEM from 3–4 mice/group.

### A Single i.t. Instillation of IL-36α does not Induce Airway Hyperresponsiveness in Mice

To determine if IL-36α induced proinflammatory effects in the lungs results in differences in physiological responses, wildtype C57BL/6 mice were intratracheally instilled with a single dose of IL-36α. Pulmonary function parameters evaluated 24 h later using invasive plethysmography revealed that the total lung resistance (Fig. 3A) and lung compliance (Fig. 3B) were not significantly different between IL-36α and PBS instilled mice.

**Figure 6:**
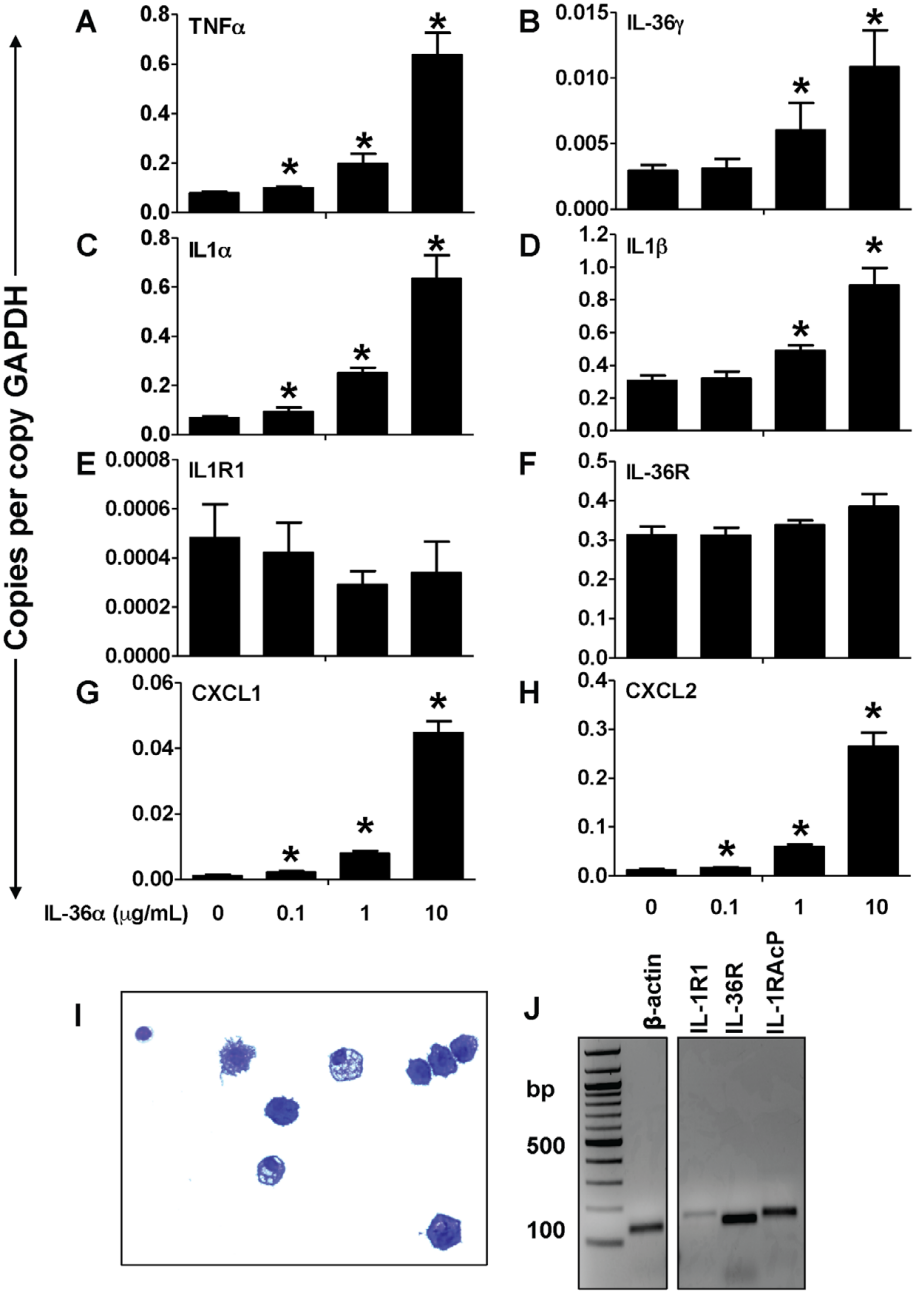
IL-36α induced the expression of proinflammatory cytokines and chemokines in splenic CD11c^+^ cells. A–H) Transcript expression of early response cytokines (TNFα, IL-1α, IL-1β, IL-36γ), the classical IL-1 receptor IL-1R1, the novel IL-1 cytokine cluster receptor IL-36R as well as the neutrophil specific chemokines CXCL1 and CXCL2 in splenic CD11c^+^ cells 2 h following incubation with increasing concentrations of IL-36α. Transcript expression was evaluated by SYBR-Green based quantitative real-time PCR. *Indicates significant differences (*P<0.05*) compared to 0 µg/mL group. Data represent mean±SD of quadruplicate samples from one of two representative experiments. I) Diff-quik stained cells from cytospun BAL cells from the lungs of naïve mice demonstrates that the majority of lung resident immune cells are alveolar macrophages. J) PCR on cDNA from naïve mouse alveolar macrophages demonstrating the constitutive mRNA expression of an endogenous control (β-actin), IL-1R1, IL-36R and IL-1RAcP. Image of a DNA electrophoresis gel has been color-inverted for clarity. bp – base pairs.

### IL-36α Induces Neutrophil Influx in the Lungs of IL-1α and IL-1β Deficient Mice

Agonist members of the IL-1 cytokine network often induce overlapping and redundant pro-inflammatory responses. For example, IL-1α and IL-1β can induce neutrophil influx into the lung and induce the production of a variety of proinflammatory mediators that are induced by IL-36α and IL-36γ. We have demonstrated that IL-36α and IL-36γ induce the production of IL-1α and IL-1β ([38] and the current study). Therefore, to determine if IL-1α and IL-1β may also contribute in part to the IL-36α induced pro-inflammatory responses in the lung, we performed a single i.t. instillation of IL-36α into the lungs of mice genetically deficient in both IL-1α and IL-1β (IL-1αβ^−/−^ mice). A single i.t. instillation of 10 µg IL-36α induced neutrophil influx (Fig. 4A) in the lungs of IL-1αβ^−/−^ mice 24 h following instillation. The majority of cells in the lungs of PBS instilled mice were macrophages. The total number of cells (Fig. 4A), percentage of neutrophils (Fig. 4B) and the total number of neutrophils (Fig. 4C) were significantly increased in the lungs of IL-1αβ^−/−^ mice that received i.t. IL-36α compared to IL-1αβ^−/−^ mice that received PBS. Flow cytometric evaluation of BAL cells also revealed that majority of cells recovered from the BAL fluid of IL-36α instilled mice were CD11c^−^CD11b^+^Ly6G^+^ neutrophils, while a majority of cells recovered from the BAL fluid of PBS instilled mice were CD11c^+^CD11b^−^Ly6G^−^ cells (Fig. 4D). Examination of hematoxylin & eosin stained lung sections also revealed significant cellular infiltration in the lungs of IL-36α instilled mice (Fig. 4E) compared to the lungs of mice that received PBS. These data demonstrate that IL-36α can induce neutrophil influx in the lungs in an IL-1α/β-independent manner.

**Figure 7:**
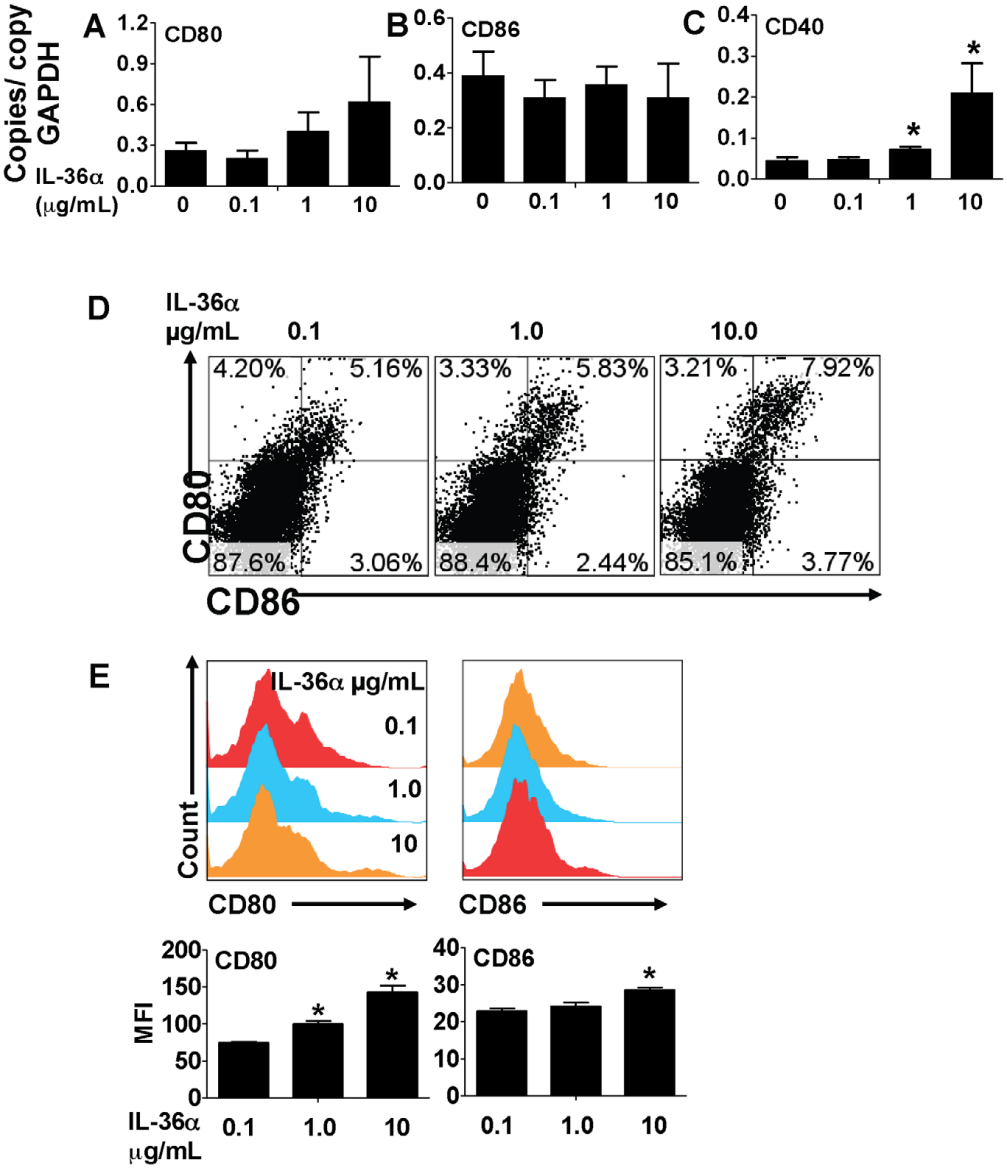
IL-36α induced the expression of T cell costimulatory molecules in splenic CD11c^+^ cells. A–C) Transcript expression of the co-stimulatory molecules CD80, CD86 and CD40 in splenic CD11c^+^ cells 2 h following incubation with increasing concentrations of IL-36α. Transcript expression was evaluated by SYBR-Green based quantitative real-time PCR. *Indicates significant differences (*P<0.05*) compared to 0 µg/mL group. Data represent mean±SD from quadruplicate samples from one of two representative experiments. D) Flow cytometric evaluation of splenic CD11c^+^ cells 24 h following incubation with increasing concentrations of IL-36α for 2 h. E) Cell surface expression of co-stimulatory molecules in splenic CD11c^+^ cells 24 h following incubation with increasing concentrations of IL-36α for 2 h. MFI – mean fluorescence intensity. *Indicates significant differences (*P<0.05*) compared to 0.1 µg/mL group. Data represent mean±SD from triplicate samples from one of two representative experiments.

**Figure 8:**
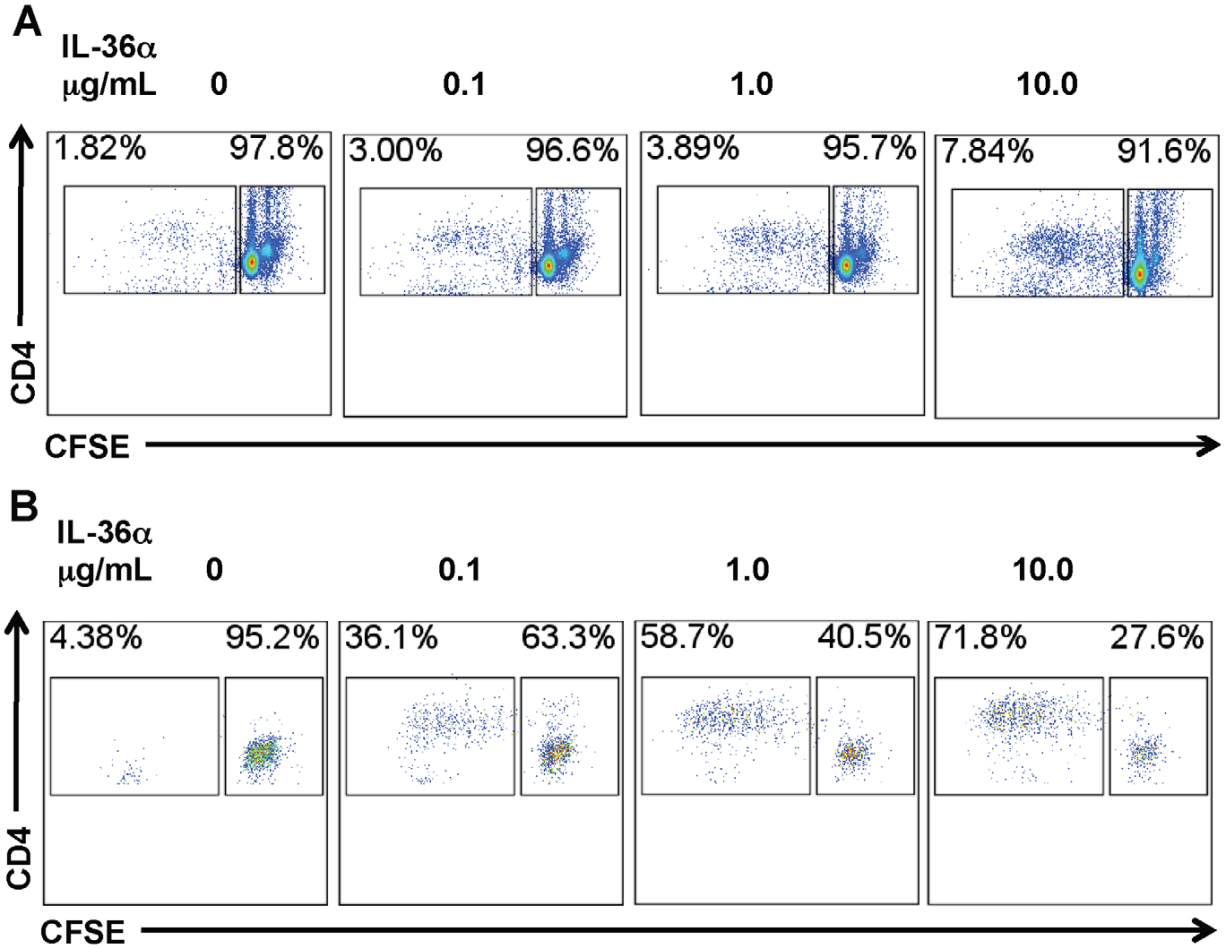
Incubation with IL-36α enhances the ability of splenic CD11c^+^ cell mediated CD4^+^ T cell proliferation. A) Flow cytometric evaluation of CD4^+^ T cell proliferation responses induced by IL-36α stimulated splenic CD11c^+^ cells. Splenic CD11c^+^ cells were incubated with increasing doses of IL-36α for 2 h. Following incubation, media containing IL-36α was removed and CFSE-labeled CD4^+^ T cells were co-cultured with IL-36α stimulated CD11c^+^ cells. CFSE dilution was used to evaluate T cell proliferation responses 96 h following co-culture. CD4^+^ T cell proliferation was proportional to the concentration of IL-36α used for stimulating CD11c^+^ cells used in the co-culture. Flow cytometry plot presented is representative of quadruplicate samples in one out of two independent experiments. B) Flow cytometric evaluation of antigen-specific CD4^+^ T cell proliferation responses induced by IL-36α stimulated splenic CD11c^+^ cells. Splenic CD11c^+^ cells were incubated with increasing doses of IL-36α and 100 ng/mL OVA_323-339_ for 2 h. Following incubation, media containing IL-36α and the OVA peptide was removed and CFSE-labeled CD4^+^ T cells from OTII TCR transgenic mice were co-cultured with IL-36α stimulated, OVA peptide pulsed CD11c^+^ cells. CFSE dilution in the culture was used to evaluate T cell proliferation responses 96 h following co-culture. CD4^+^ T cell proliferation was proportional to the concentration of IL-36α used for stimulating CD11c^+^ cells used in the co-culture. Flow cytometry plot presented is representative of quadruplicate samples in one out of two independent experiments.

### IL-36α Induces the Expression of Early Response Cytokines and Neutrophil Specific Chemokines in the Lungs of IL-1α and IL-1β Deficient Mice

To determine if IL-36α induced cytokine and chemokine expression is altered in the absence of IL-1α and IL-1β, we collected lung tissue from IL-1αβ^−/−^ mice 24 h following a single i.t. instillation of IL-36α and profiled the mRNA expression of selected cytokines, chemokines as well as agonists and receptors in the IL-1 cytokine network. The mRNA expression of the early response cytokine TNFα (Fig. 5A) as well as IL-36γ Fig. (5B) was significantly increased in the lungs of IL-1αβ^−/−^ mice i.t. instilled with IL-36α, compared to saline controls. As expected, mRNA expression of neither IL-1α nor IL-1β was detected in the lungs of IL-1αβ^−/−^ mice (data not shown). While the mRNA expression of the classical IL-1 receptor (IL-1RI) was unchanged (Fig. 5C), the mRNA expression of IL-36R was significantly increased in the lungs of IL-36α instilled IL-1αβ^−/−^ mice (Fig. 5D). In addition, the mRNA expression of the neutrophil recruiting chemokines CXCL1 (Fig. 5E) and CXCL2 (Fig. 5F) were also increased in the lungs of IL-36α instilled IL-1αβ^−/−^ mice compared to PBS controls. While TNFα protein levels were increased in the BAL fluid recovered from the lungs of IL-36α instilled IL-1αβ^−/−^ mice, the increase was not statistically significant (Fig. 5G). However, the protein levels of CXCL1 were significantly increased in the BAL fluid recovered from the lungs of IL-36α instilled IL-1αβ^−/−^ mice (Fig. 5H). These results suggest that IL-36α can induce the expression of its receptor (IL-36R) and the neutrophil chemokines CXCL1 and CXCL2 in the absence of both IL-1α and IL-1β.

**Figure 9:**
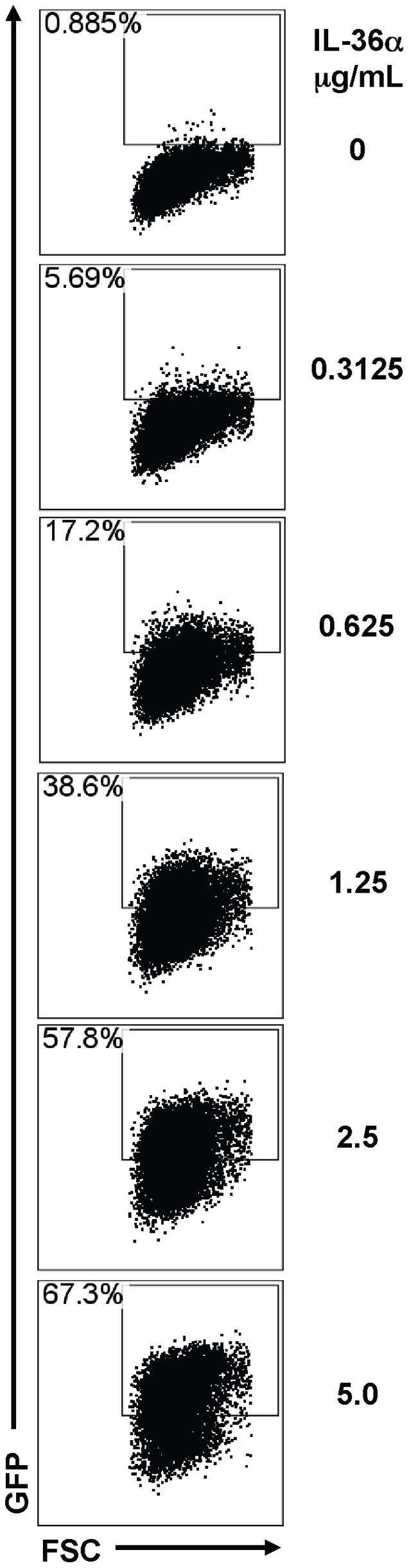
IL-36α directly induced NF-κB activation in mouse macrophage cell lines. A) Cells from a mouse macrophage NF-κB reporter cell line (RAW-ELAM cells) were stimulated with increasing concentrations of IL-36α. Green fluorescent protein (GFP) expression, indicative of NF-κB activation, was increased in a dose-dependent manner upon incubation with IL-36α. Flow cytometry plot presented is representative of triplicate samples in one out of two independent experiments.

### IL-36α Induces the mRNA Expression of Early Response Cytokines and Neutrophil-specific Chemokines in Splenic CD11c^+^ Cells

IL-36α and IL-36γ have been reported to be expressed and inducibly upregulated in airway epithelial cells in response to various stimuli [39]. In addition, recent reports demonstrated that bone marrow derived dendritic cells express IL-36R, the receptor for IL-36α and IL-36γ [51]. Since more than 95% of immune cells in a naïve mouse lung are CD11c^+^ alveolar macrophages, we hypothesized that under inflammatory conditions, overproduced IL-36α may activate CD11c^+^ cells, leading to the production of inflammatory mediators that orchestrate the cellular immune responses in the lung. To determine if IL-36α has direct effects on cytokine production and antigen-presenting abilities of CD11c^+^ cells, we first isolated splenic CD11c^+^ cells and incubated them with increasing concentrations of IL-36α for 2 h. The mRNA expression of TNFα (Fig. 6A), IL-36γ (Fig. 6B), IL-1α (Fig. 6C) and IL-1β (Fig. 6D) were increased in a dose-dependent manner upon incubation with IL-36α. However, the expression of neither IL-1RI (Fig. 6E) nor IL-36R (Fig. 6F) was increased in CD11c^+^ cells upon incubation with IL-36α. It is important to note that the baseline expression of IL-36R in CD11c^+^ cells was relatively high (∼0.3 copies/copy GAPDH, Fig. 6F), suggesting that CD11c^+^ cells may readily respond to IL-36α and IL-36γ. In addition, IL-36α also induced a dose-dependent increase in the mRNA expression of neutrophil recruiting chemokines such as CXCL1 (Fig. 6G) and CXCL2 (Fig. 6H) in CD11c^+^ cells. These data also demonstrate that IL-36α directly acts on CD11c^+^ cells to induce the production of neutrophil recruiting chemokines, elucidating a molecular mechanism that potentially drives the influx of neutrophils to the lung following i.t. instillation of IL-36α. While the results described above were obtained using splenic CD11c^+^ cells, we also demonstrate that alveolar macrophages isolated from the lungs of naïve wild-type mice (Fig. 6I) express IL-1R1, IL-36R and IL-1RAcP (Fig. 6J). In addition to previous reports demonstrating IL-36R expression in BMDCs and T cells, here we show that alveolar macrophages also have the molecular machinery to respond to IL-36 cytokines. Further studies are required to determine if other cells that infiltrate the lung during an immune response also express IL-36R.

### IL-36α Induces the Expression of co-stimulatory Molecules in Splenic CD11c^+^ Cells and Enhances CD4^+^ T Cell Proliferation

Activation and excessive proliferation of T cells is a critical component in the pathogenesis of a multitude of inflammatory disorders, including lung disorders such as asthma. A recent report demonstrated that CD4^+^ T cells and CD11c^+^ cells express IL-36R, the receptor for IL-36α and the other IL-36 cytokines [51]. To determine if IL-36α regulated T cell proliferation by modulating costimulatory molecule expression in APCs, we incubated splenic CD11c^+^ cells with increasing concentrations of IL-36α and measured the mRNA expression of costimulatory molecules at earlier timepoints and cell surface expression of the costimulatory molecules at the later timepoints. While incubation of CD11c^+^ cells with IL-36α did not induce the mRNA expression of CD80 (Fig. 7A) or CD86 (Fig. 7B) in CD11c^+^ cells 2 h following incubation, the expression of the co-stimulatory molecule CD40 was significantly increased in a dose-dependent manner (Fig. 7C). Incubation of splenic CD11c^+^ cells with IL-36α for 2 h was sufficient to induce a modest, but dose-dependent increase in the percentage of CD80^+^CD86^+^ cells 24 hours later (Fig. 7D). Furthermore, incubation of splenic CD11c^+^ cells with IL-36α for 2 h also significantly increased cell surface CD80 expression and a modest, but significant increase in cell surface CD86 expression 24 hours later (Fig. 7E). In addition, the proliferation of CD4^+^ T cells was proportionally increased when co-cultured with CD11c^+^ cells pre-incubated with increasing concentrations of IL-36α (Fig. 8A). Antigen-specific CD4^+^ T cell proliferation was also proportionally increased when CD4^+^ T cells from OVA-specific OTII TCR transgenic mice were co-cultured with CD11c^+^ cells pre-incubated with increasing concentrations of IL-36α and a fixed concentration of OVA_323–339_ peptide, the cognate antigen. Since the IL-36α containing media was removed from the CD11c^+^ cells before addition of CD4^+^ cells, these data suggest that IL-36α induced expression of costimulatory molecules or mediators may indirectly contribute to T cell proliferation responses.

### IL-36α Induces the Activation of NF-κB in Mouse Macrophage Cell Lines

Previous studies have reported that agonist IL-36 cytokines such as IL-36α and IL-36γ induce NF-κB activation in Jurkat cells transfected with IL-36R [31]. We have previously demonstrated that IL-36γ induces the activation of NF-κB in macrophage cell lines in a dose-dependent manner [38]. Here, we demonstrate that similar to IL-36γ, IL-36α also induces the activation of NF-κB in a mouse macrophage cell line (Fig. 9). These data suggest that IL-36α induced NF-κB activity may be a critical mechanism in driving the production of proinflammatory mediators by macrophages and proliferation of T cells by enhancing the stimulatory properties of dendritic cells.

## Discussion

The current study demonstrates that IL-36α acts as a pro-inflammatory cytokine in the lungs independent of both IL-1α and IL-1β. Intratracheal instillation of IL-36α induced neutrophil influx and increased the expression of pro-inflammatory cytokines and chemokines in the lungs of wild-type C57BL/6 mice as well as mice deficient in both IL-1α and IL-1β. IL-36α acted directly on CD11c^+^ antigen presenting cells to induce the production of early response cytokines and neutrophil-specific chemokines. In addition, IL-36α increased the expression of T cell co-stimulatory molecules on CD11c^+^ cells and enhanced their ability to stimulate CD4^+^ T cell proliferation. Stimulation of a macrophage cells line with IL-36α induced NF-κB activation. Collectively, these data suggest that disease-induced overproduction of IL-36α in the lungs may play an important role in inflammatory lung disease.

Intratracheal instillation of IL-36α induced neutrophil influx in the lungs of wildtype and IL-1αβ^−/−^ mice. Transgenic mice that conditional overexpress IL-1β in the airway epithelium have neutrophil influx, increased mucus production and the development of emphysematous and fibrotic changes in the lung [52]. Prior reports also demonstrated that intratracheal administration of IL-1 induced neutrophil influx into the lungs of mice [53], and that IL-1 is necessary for lipopolysaccharide (LPS) mediated neutrophil influx in the murine lung [54], [55]. We previously reported that intratracheal administration of IL-36γ also induced neutrophil influx into the lungs and the expression of neutrophil-specific chemokines [38]. Here we show that IL-36α also resulted in neutrophil chemotaxis in the lungs, along with an increase in the mRNA expression of the classical IL-1 agonists IL-1α and IL-1β in wild-type mice. This supports our previous findings [38] and those of others [39] demonstrating that agonist IL-36 cytokines induce the production of classical IL-1 cytokines. Importantly, in the current study intratracheal instillation of IL-36α induced neutrophil influx into the lungs of IL-1αβ^−/−^ mice, suggesting that IL-36α driven neutrophil recruitment to the lungs is independent of IL-1α and IL-1β.

The mRNA expression of early response cytokines such as TNFα, IL-1α and IL-1β were increased in the lungs of wildtype mice following intratracheal instillation of IL-36α. These results are consistent with our previous reports demonstrating that i.t. instillation of IL-36γ into the lungs of mice induced the production of IL-1α in a dose-dependent manner [38]. While the mRNA expression of IL-1R1 was unchanged, the expression of IL-36R was significantly increased in the lungs of IL-36α treated mice, demonstrating that IL-36α can induce the expression of its own receptor in lung tissue either by directly acting on structural or immune cells in the lung, or indirectly by inducing the production of other mediators in the lung that increase IL-36R expression. Since the expression of IL-36R was also induced in the lungs of IL-36α instilled IL-1αβ^−/−^ mice, it is possible to speculate that neither IL-1α nor IL-1β contribute to IL-36α driven IL-36R expression. IL-1 induced the mRNA expression of multiple chemokines in airway epithelial cells *in vitro* [56]–[58] and *in vivo* [52]. Furthermore, overexpression of IL-1β in the airway epithelium increased the mRNA expression of CXCL1, CXCL2, CCL2 and CCL7 chemokines and the accumulation of neutrophils in the lungs of mice [59]. Increased mRNA expression of CXCL1 and CXCL2 in the lungs of IL-36α instilled wildtype as well as IL-1αβ^−/−^ mice in the current study suggests that IL-36α can induce the expression of neutrophil-specific chemokines in the lungs independent of IL-1α and IL-1β.

We have previously demonstrated that a single intratracheal instillation of IL-36γ induces airway hyperresponsiveness in response to aerosolized methacholine [38]. However, in the current study, a single i.t. instillation of a similar amount of IL-36α did not increase airway hyperresponsiveness. This discrepancy could be due to inherent differences in the ability of IL-36α and IL-36γ in inducing AHR. For example, we have shown that unlike IL-36γ, IL-36α is not constitutively expressed in lungs [38]. Furthermore, the magnitude of induction of IL-36α mRNA in response to cytokines and other inflammatory stimuli appears to be consistently lower than that of IL-36γ mRNA expression in airway epithelial cells [39], bone marrow derived dendritic cells (BMDCs) and T cells [51]. Therefore, it is possible that a higher concentration of IL-36α is required to produce similar magnitude of responses driven by IL-36γ at a lower concentration. In addition, the discrepancy could also be due to differences in AHR-specific genetic susceptibilities between A/J mice used in our previous report [38] and the C57BL/6 strain of mice used in this study.

IL-36α acted directly on splenic CD11c^+^ cells to induce the production of neutrophil-specific chemokines. A recent report demonstrated that IL-36R was expressed in murine BMDCs and CD4^+^ T cells and that the stimulation of BMDCs with IL-36α, IL-36β and IL-36γ induced the production of multiple cytokines and chemokines [51]. In the current study we demonstrate that IL-36α acts directly on splenic CD11c^+^ cells to induce the production of the neutrophil-specific chemokines CXCL1, CXCL2 as well as other early response cytokines such as TNFα, IL-1α and IL-1β. Interestingly, the mRNA expression of neither IL-1R1 nor IL-36R was increased in IL-36α stimulated splenic CD11c^+^ cells under the conditions tested. Since we observed increased IL-36R expression in whole lung tissue following a single IL-36α challenge, we speculate that IL-36α either directly increases IL-36R in non-CD11c^+^ cells in the lung, or induces the production of another endogenous mediator from an IL-36α responsive, non-CD11c^+^ cell type in the lungs to increase IL-36R mRNA expression *in vivo*. Furthermore, we have also demonstrated that similar to splenic CD11c^+^ cells, alveolar macrophages from naïve mice also express IL-36R mRNA at levels higher than IL-1R1, suggesting that alveolar macrophages are poised to respond to IL-36 cytokines released into the lung airspaces.

IL-36α acted directly on CD11c^+^ cells to induce the expression of co-stimulatory molecules that regulate T cell activation and proliferation. While activation and proliferation of T cells are essential to confer protection against certain pathogens, aberrant activation and proliferation of T cells leads to detrimental responses in lung diseases such as asthma [60]. Apart from T cell receptor activation, engagement of co-stimulatory molecules expressed on T cells (such as CD28 and CD40L) with those expressed on antigen presenting cells (such as CD80, CD86 and CD40) are critical for T cell activation and proliferation [61]. Previous reports have demonstrated that IL-1β induces antigen-specific proliferation of CD4+ T cells during the primary and secondary (memory) immune responses [62], and that CD4^+^ T cells from IL-1αβ^−/−^ mice are defective in proliferation and the production of T helper 2 (Th2) cytokines [13]. It has also been demonstrated that IL-1α induced CD40 expression in human dendritic cells *in vitro* [63]. Consistent with a recent report [51], we also found that IL-36α directly induced the mRNA expression of CD40 in CD11c^+^ cells. Although both CD80 and CD86 mRNA were constitutively expressed in CD11c^+^ cells in our experiments, mRNA expression was not significantly increased 2 h following stimulation with IL-36α. However, we found that the cell surface expression of CD80 and CD86 were increased in splenic CD11c^+^ cells 24 hours following 2 hours of stimulation with IL-36α. We also found that CD4^+^ T cell proliferation responses were enhanced when co-cultured with IL-36α stimulated CD11c^+^ cells, suggesting that IL-36α mediated upregulation of costimulatory molecules may be important for T cell proliferation. Interaction of CD40 with CD40L is important for Th1 cell differentiation [64]. Vigne et al., recently demonstrated that IL-36β (IL-1F8) increased CD40 mRNA expression and enhanced Th1 responses *in vivo* [51]. Collectively, these data support an important role of novel IL-1 cytokine agonists in regulating T cell activation and proliferation by modulating antigen presentation and co-stimulatory abilities of APCs. Experiments in the current study were performed using splenic CD11c^+^ cells, which may have different cytokine secretion and antigen-presenting capabilities than CD11c^+^ cells that reside in or are recruited to the lungs during pulmonary inflammation [65]. We have shown that alveolar macrophages isolated from naïve wild-type mice express IL-36R and IL-1RAcP, suggesting that alveolar macrophages are poised to respond to IL-36 cytokines in the lung. While we speculate that the responses of lung CD11c^+^ cells to IL-36α would be largely similar to the responses reported in the current study, further studies are required to determine the functional effects of IL-36α on lung specific CD11c^+^ cells and subsets.

IL-36α activated NF-κB in mouse macrophages. Activation of NF-κB in the lungs leads to airway inflammation, mucus cell metaplasia and the production of proinflammatory cytokine and chemokines [66]–[68]. Agonist members of the novel IL-1 cytokine cluster induced NF-κB and MAPK activation in Jurkat cells transfected with IL-36R [31]. We previously demonstrated that IL-36γ induced NF-κB activation in mouse macrophages following *in vitro* stimulation and in total lung tissue following a single intratracheal challenge with IL-36γ [38]. In addition, IL-36γ also induced NF-κB activation in lung fibroblasts [39]. Consistent with these findings, IL-36α also induced NF-κB activation in mouse macrophages, which may be a potential mechanism driving the production of neutrophil-specific chemokines from macrophages and dendritic cells in the lung resulting in recruitment of neutrophils. Furthermore, these results also confirm previous findings that NF-κB is a critical signaling molecule downstream of both IL-1R1 and IL-36R mediated responses.

To date, the role of IL-36α has not been examined in human inflammatory lung disease. It is interesting that very high concentrations of the IL-36 agonists including IL-36α are required to stimulate *in vitro* cellular responses [30], [31]. A recent report demonstrates that truncation variants of IL-36α, IL-36β and IL-36γ exert enhanced agonist activity *in vitro*, suggesting that a yet unidentified protease may process these cytokines to more biologically potent forms *in vivo* [69]. Although the magnitude of the cellular responses was enhanced by the truncation variants, the underlying IL-36 cytokine driven mechanisms such as NF-κB activation remain similar. Therefore, we conclude that although that the results reported in this study using the full-length IL-36α reflect the functional consequences of IL-36α overproduction in the lung, further studies are required to determine if the truncated versions of IL-36α proteins would induce more severe inflammation in the lung *in vivo*. Furthermore, since the IL-36 cytokine agonists do not possess a leader peptide that is necessary for cytokine secretion [70], the *in vivo* mechanisms by which these cytokines are released into the extracellular spaces such as BAL fluid remains to be clarified. While there are several reports demonstrating increased mRNA expression of novel IL-1 cytokine agonists, there is a paucity of reports on the *in vivo* detection of the cytokines at the protein level in extracellular spaces. Among the several mechanisms by which IL-1β has been proposed to become available in the extracellular space [71]–[75], plasma membrane breakdown has been suggested to be the most plausible mechanism by which intracellular IL-36α could become available in the extracellular space [76]. The current study determined that IL-36α acts as a pro-inflammatory cytokine in the lung, and that IL-36α may increase inflammatory responses in disease conditions which involve release of IL-36α into the lungs.

## Supporting Information

Figure S1 Intratracheal instillation of IL-36α induced neutrophil influx in the lungs of endotoxin resistant C3H/HeJ mice. A) Total cell counts from BAL fluid recovered from mice 24 h following a single i.t. instillation of PBS or 10 µg IL-36α. C) Differential cell count percentages and D) Differential cell count numbers in the BAL fluid recovered from mice 24 h following a single i.t. instillation of PBS or 10 µg IL-36α. *Indicates significant differences (*P<0.05*) compared to PBS treated mice. Data represent mean±SEM from 4–5 mice/group.
(TIFF)
